# Embedding routine health care data in clinical trials: with great power comes great responsibility

**DOI:** 10.1007/s12471-023-01837-5

**Published:** 2024-01-15

**Authors:** M. Louis Handoko, Frances S. de Man, Jasper J. Brugts, Peter van der Meer, Hanneke F. M. Rhodius-Meester, Jeroen Schaap, H. J. Rik van de Kamp, Saskia Houterman, Dennis van Veghel, Alicia Uijl, Folkert W. Asselbergs

**Affiliations:** 1 Department of Cardiology, Amsterdam University Medical Centres, Amsterdam, The Netherlands; 2 Heart Failure and Arrhythmias, Amsterdam Cardiovascular Sciences, Amsterdam, The Netherlands; 3 Department of Pulmonary Medicine, Amsterdam University Medical Centres, Amsterdam, The Netherlands; 4 Amsterdam Cardiovascular Sciences, Pulmonary Hypertension and Thrombosis, Amsterdam, The Netherlands; 5 Department of Cardiology, Thorax Centre, Erasmus Medical Centre, University Medical Centre Rotterdam, Rotterdam, The Netherlands; 6 Department of Cardiology, University Medical Centre Groningen, University of Groningen, Groningen, The Netherlands; 7 Department of Internal Medicine, Geriatrics Section, Amsterdam University Medical Centres, Amsterdam, The Netherlands; 8 Department of Neurology, Alzheimer Centre Amsterdam, Amsterdam University Medical Centres, Amsterdam, The Netherlands; 9 Department of Geriatric Medicine, Oslo University Hospital, Ullevål, Oslo, Norway; 10 Department of Cardiology, Amphia Hospital, Breda, The Netherlands; 11 Dutch Network for Cardiovascular Research, Utrecht, The Netherlands; 12 Netherlands Heart Registration, Utrecht, The Netherlands; 13 Julius Centre for Health Sciences and Primary Care, University Medical Centre Utrecht, Utrecht, The Netherlands; 14 Division of Cardiology, Department of Medicine, Karolinska Institutet, Stockholm, Sweden; 15 Institute of Cardiovascular Science and Institute of Health Informatics, Faculty of Population Health Sciences, University College London, London, UK

**Keywords:** Randomised controlled trials, Pragmatic clinical trials, Big data, Cardiovascular diseases, Heart failure

## Abstract

Randomised clinical trials (RCTs) are vital for medical progress. Unfortunately, ‘traditional’ RCTs are expensive and inherently slow. Moreover, their generalisability has been questioned. There is considerable overlap in routine health care data (RHCD) and trial-specific data. Therefore, integration of RHCD in an RCT has great potential, as it would reduce the effort and costs required to collect data, thereby overcoming some of the major downsides of a traditional RCT. However, use of RHCD comes with other challenges, such as privacy issues, as well as technical and practical barriers. Here, we give a current overview of related initiatives on national cardiovascular registries (Netherlands Heart Registration, Heart4Data), showcasing the interrelationships between and the relevance of the different registries for the practicing physician. We then discuss the benefits and limitations of RHCD use in the setting of a pragmatic RCT from a cardiovascular perspective, illustrated by a case study in heart failure.

## Introduction

In the hierarchy of clinical evidence, well-executed randomised clinical trials (RCTs) are considered to be at the top of the evidence pyramid; however, large phase-3 RCTs are very complex endeavours. They are expensive and inherently slow. Also, the generalisability of RCTs has been questioned, with the underrepresentation of women, the frail elderly and the underprivileged.

Observational analyses of routine health care data (RHCD), derived from very large electronic patient databases, have been proposed as an attractive alternative. Unfortunately, these non-randomised analyses often do not suffice, or may even be misleading in evaluating the efficacy and safety of interventions [1]. Since the beneficial effects of treatments are usually modest, randomisation is a prerequisite for a reliable assessment. Nonetheless, there is considerable overlap in RHCD and trial-specific data. Integration of RHCD in an RCT has great potential, as it would reduce the effort and costs required to collect data, thereby overcoming some of the major downsides of a ‘traditional’ RCT.

This viewpoint first explains what RHCD encompasses, and provides a current overview of cardiovascular registries in the Netherlands (Netherlands Heart Registration (NHR), Heart4Data). Subsequently, the benefits and challenges of using RHCD in an RCT are discussed from a cardiovascular perspective, illustrated by a case study in heart failure.

## What are RHCD and what is already available in The Netherlands?

**Fig. 1:**
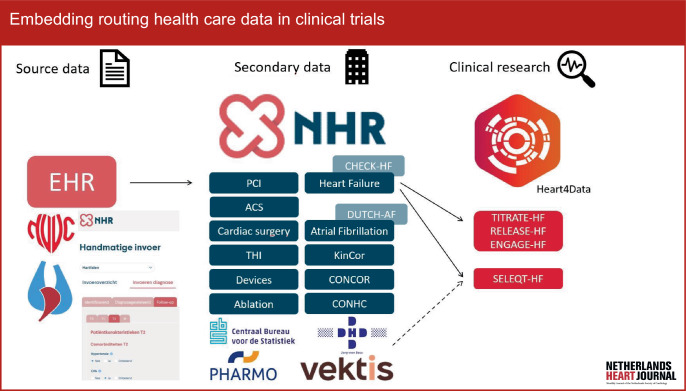
Overview of routine health care (or source) data from electronic health care records (*EHR*) of cardiology clinics in the Netherlands (represented by the Dutch Society of Cardiology and the Dutch Society of Cardiothoracic Surgeons) and secondary data that are relevant to the Dutch cardiologist. Secondary data consist of the different registries coordinated and managed by the Netherlands Heart Registration (*NHR*), but also comprises data collected by, for example, Statistics Netherland (*Centraal Bureau voor de Statistiek*), DHD (registries from hospitals), Pharmo (nationwide database of drug descriptions from various primary and secondary health care settings) and Vektis (which handles all health insurance claims in the Netherlands). The Dutch CardioVascular Alliance Heart4Data consortium aims to develop a sustainable infrastructure for cardiovascular registry-based research in the Netherlands, including governance and an information technology infrastructure, research methods, FAIR (findable, accessible, interoperable and reusable) data creation and data linkage with relevant databases. Heart4Data focuses on the NHR Heart Failure and Atrial Fibrillation registries, because these are chronic conditions with a major clinical impact. The NHR Heart Failure registry is the successor of CHECK-HF; the NHR Atrial Fibrillation registry is the successor of DUTCH-AF. TITRATE-HF and associated trials (ENGAGE-HF, RELEASE-HF) are observational studies and act as accelerators of the NHR Heart Failure registry. SELEQT-HF will be the first registry-based randomised clinical trial to make use of the NHR Heart Failure registry infrastructure (complemented by other data sources). *PCI* percutaneous coronary intervention, *ACS* acute coronary syndrome, *THI* transcatheter heart valve intervention. *KinCor, CONCOR *and *CONHC* are registries for paediatric and adult patients, respectively, with a congenital heart disease

RHCD refers to large datasets that contain health-related information primarily collected for clinical reasons, as opposed to trial-specific data [2]. These may comprise source data (medical notes, laboratory results, images) and/or secondary data (diagnostic codes, insurance claims, quality registries). Thus, every practicing physician and nurse generates large amounts of valuable data every day that could be repurposed for goals beyond direct patient care, such as quality control, implementation of new guidelines and clinical research. National registries that collect data on hospitalisation or mortality are other examples of RHCD. In the Netherlands, we already have a wide array of cardiovascular registries, with the BHN (*Begeleidingscommisie Hartinterventies Nederland*) registry for cardiac surgery being one of the first (since 1993). In October 2017, in collaboration with the professional societies (Dutch Society of Cardiology (NVVC); Dutch Society of Cardiothoracic Surgeons (NVT)), three separate quality registries merged into one national registry: the Netherlands Heart Registration (NHR) (Fig. 1; [3]), with registries on atrial fibrillation and heart failure being some of its most recent additions [4]. RHCD are certainly not new, but became more widely available—and therefore more relevant—for secondary analyses following the widespread introduction of electronic health records. The potential of RHCD for clinical research is immense. To illustrate this fact, the electronic health records of the National Health Service (NHS) contain a longitudinal medical history of 98% of the UK population (67 million people), and include over 900,000 heart failure patients [2]. The NHR already covers over 1.5 million cardiac procedures, and over 80,000 procedures are added yearly [3]. To unleash the scientific potential of current Dutch cardiovascular registries, the Heart4Data consortium was established under the wing of the Dutch CardioVascular Alliance (DCVA) [5].

## Potential benefits of using RHCD in randomised clinical trials

### Larger trials

Use of RHCD may be of assistance at all stages of an RCT. Foremost, successful integration of RHCD in the setting of an RCT will significantly reduce the effort and costs required to collect data. Consequently, this allows for an increase in the number, size and speed of trials. Furthermore, very large trials permit advanced designs such as adaptive platform trials, which could accelerate the generation of knowledge even further. A prime example of such a trial is RECOVERY [6]. In just 2 years after it began in March 2020, nearly 50,000 patients hospitalised for COVID were recruited, and the efficacy and safety of nine compounds have already been reliably evaluated.

### Better-informed trial design

Exploration of RHCD may reveal heterogeneity in disease management, which may evoke novel research questions. In heart failure with reduced ejection fraction, for example, the most recent guideline of the European Society of Cardiology (ESC) proposed initiating the four classes of drugs that lower morbidity and mortality (i.e. angiotensin receptor blocker/neprilysin inhibitor, beta-blocker, mineralocorticoid receptor antagonist, sodium-glucose cotransporter 2) as early as possible [7]. The guideline also prioritises introduction (at a low dose) of different drug classes over dosage up-titration. The recommendation diverges from previous guidelines that advocated a sequential approach. Interestingly, this new recommendation was motivated by literature that was previously used to argue in favour of the opposite standpoint [8]. Although a modelling study suggests that the new approach has an impressive beneficial effect on survival [9], prospective clinical evidence from a large RCT is limited [10]. An observational analysis of RHCD, comparing the effect of the new and old approach, can estimate the potential clinical benefit, which in turn may guide sample size calculation for such a trial.

Furthermore, RHCD may be very helpful in assessing the feasibility of a trial. With RHCD, the effect of adjustments of inclusion and exclusion criteria on the pool of potentially eligible study participants can be assessed directly.

### Enhanced recruitment of trial participants

In line with optimising feasibility, recruitment could also be enhanced by RHCD. Once identified, ideally by ‘nudging’ (a system-based alert system that makes real-time use of RHCD), potential study participants can be approached directly by the coordinating research site. In the past, the ASCEND trial (aspirin and/or omega‑3 fatty acid supplements for the primary prevention of cardiovascular events in people with diabetes) was able to recruit over 15,000 patients by direct mail after identification of eligible patients using national and local diabetes registries [11].

### More complete and longer trial follow-up

Follow-up that is complete and sufficiently long is key to reliably assessing the efficacy and safety of an intervention studied. In general, trial follow-up is labour-intensive and therefore costly. Also, extensive patient engagement is required, which may be particularly troublesome for typically underrepresented demographic groups like the elderly or underprivileged. Minimising the efforts required of the study team, patients and caregivers by smart integration of RHCD will undoubtedly improve the quality of follow-up. As a bonus, a longer follow-up is possible with only minor additional work. An important caveat is nationwide access to RHCD to prevent incomplete follow-up and underreporting. Again, the ASCEND trial is a prime example [10]. At the end of the trial, access was granted to UK hospital episode statistics (HES), which complemented the trial-specific mail-based follow-up by self-reporting. The investigators were able to report on the effect of aspirin on dementia prevention with a median follow-up of 7.4 years, whereby 99% of the participants could be linked to UK HES [12].

## Challenges and limitations of RHCD in clinical trials

### Data protection, privacy, informed consent

Embedding RHCD in RCT has great potential, as explained in the previous section. However, ‘with great power comes great responsibility’. Privacy and confidentiality are core principles of a safe patient-physician relationship. Electronic health records have made sensitive medical information relatively easily accessible, but the same holds for malicious parties, too. Data protection, therefore, is more relevant than ever. Databases must comply with international frameworks for information security. In 2018, a new European law on privacy, the General Data Protection Regulation, came into force (Dutch: *Algemene Verordening Gegevensbescherming*). This law acknowledges that secondary analyses of RHCD are of interest to the public but, at the same time, the law is very strict on which data are allowed to be processed. The collection and use of personal data are only acceptable when patient data are used for monitoring quality of care. However, if data are used for medical research, a stricter legal regime must be followed [13]. The law does not consider pseudonymised data to be anonymous, and in these cases informed consent is required; a consent waiver may only apply in exceptional cases. However, the standard method to obtain informed consent via an opt-in procedure, whereby each person is explicitly asked for permission in advance, is considered highly impractical by the research community, and therefore a public debate is currently taking place.

Fortunately, the law provides some leeway for informed consent via the opt-out procedure, whereby a person is informed that their data may also be used for (observational) medical research and is reminded of their right to revoke their consent. A few RCTs have compared the traditional opt-in to the opt-out approach in order to obtain informed consent for (research) registries [14]. As expected, the participation rate using the opt-out method was much higher (96% vs 21%). More importantly, the population in the opt-out group was more representative [15]. An additional survey confirmed that patients and caregivers support the opt-out approach and prefer it over the opt-in method [16]. Also, the (research) registries that obtain informed consent by the opt-out approach are generally of higher quality than their counterparts [17]. It therefore seems reasonable to use health records for observational medical research, provided the public is informed and offered the choice of opting out, in order to comply with the current privacy legislation. For clarification, if patients are identified by RHCD, informed consent by the opt-in approach remains the preferred method before randomisation into an RCT.

Finally, there is the issue of access to the data. Public trust in medical research must always be upheld. As a general rule, it is currently not possible to directly invite potentially eligible patients to participate in a study if there is no previous treatment relationship. To overcome this barrier, it would be prudent if a dedicated, independent medical ethics committee could consider applications for waiving consent in order to invite people to participate. Such a committee would have to carefully weigh the benefits against other ethical values such as autonomy, fidelity and justice [18].

### Data linkage: technical and practical issues

RHCD cannot be used for RCTs if the variables of interest are not routinely evaluated. For example, quality-of-life questionnaires are not structurally assessed, which may hamper cost-effectiveness analysis. On the other hand, by linkage, several different sources of data (preferably discrete data instead of free text) can be combined to enrich the data with multiple facets, making RCHD use in RCTs a very powerful approach. Ideally, a unique identifier (e.g. social security number) is used in combination with several strong identifiers (e.g. name, date of birth). Also, linkage of different systems may be necessary to accomplish nationwide coverage.

Nonetheless, different data formats and inconsistencies within datasets may severely complicate linkage [19]. In addition, data are owned and managed by different parties who may not be inclined to collaborate. Finally, due to limited resources data linkage simply has low priority. However, at a time of crisis, and with the coordinating help of the Health Data Research Hub for Clinical Trials, RECOVERY was able to link 25 different registries in record time [20]. This demonstrates that ‘where there’s a will, there’s a way’. There are also successful examples of registry linkage in The Netherlands, despite its decentralised organisation [21, 22].

### Is the quality of RCHD sufficient for use in RCT?

It is fair to question the quality of RCHD, since—by definition—data are not collected with the specifics of clinical trials in mind. Depending on the source of RCHD, missing data, misclassification, underreporting and overreporting are possible causes of reduced accuracy. However, if errors are random and datasets sufficiently large, RCHD are surprisingly robust as regards bias by the ‘magic of randomisation’ [1].

In ASCEND, outcome measures from UK HES were validated against trial-specific, adjudicated data [22]. The primary outcome was a composite of different severe cardiovascular events (i.e. non-fatal myocardial infarction, ischaemic stroke, transient ischaemic attack, vascular cardiovascular death, excluding haemorrhagic stroke). Overall, there was underreporting of events (1009 vs 1401). Nonetheless, the investigator observed a strong agreement between the two methods of follow-up (kappa: 0.78; a kappa value > 0.75 represents excellent agreement), and consequently the rate ratios for the aspirin-randomised comparison did not differ for either method. Interestingly, the extent of agreement was very high among the different components of the primary endpoint (kappa values varied between 0.73 and 0.94), except for transient ischaemic attacks (kappa: 0.43). From a clinical perspective, transient ischaemic attacks are relatively poorly defined, which may explain the modest agreement.

Heart failure is also a clinical syndrome where variable clinical presentation may undermine consistent classification. In addition, classification is further complicated by the changing nomenclature over time. Blecker et al. evaluated different automated algorithms to identify heart failure cases in a large local dataset containing almost 50,000 hospitalisations [23]. When relying on structured data, the positive predictive value for identifying heart failure events was high (96%) but sensitivity was poor (40%). However, when machine learning techniques and natural language processing were used to analyse data, sensitivity more than doubled (83%) whereas the positive predictive value remained acceptable (90%). The findings demonstrate that advanced algorithms could significantly increase the usefulness of RHCD, but are probably more difficult to implement at a national level.

In summary, the quality of RHCD is sufficient to capture clinical events, noting that external validation is necessary before use. Underreporting by RHCD is observed, particularly when the clinical event of interest cannot be clearly defined. (Very) large datasets can compensate for underreporting and randomisation makes them almost impervious to bias.

### Consequence of time lag

RCHD are usually significantly time-lagged, and this is especially true for secondary care records such as health insurance claims, where coding is typically done by non-clinicians, weeks after the event. This may not be a major issue for interim analyses performed by a data monitoring committee; however, for pharmacovigilance or reporting on intervention or device-related adverse events, RCHD will most likely not suffice.

Nevertheless, there are exceptional cases where RCHD did suffice for rapid safety reporting. One example is the Salford Lung Study, in which the efficacy and safety of fluticasone/vilanterol inhalation was evaluated against standard of care in a primary care setting [24]. Patients were continuously monitored via real-time data collection from general practices and hospitals, among other systems. A safety alerting and reporting system was established based on serious adverse events, being initially flagged in the electronic health record. This would then prompt rapid evaluation and safety reporting if appropriate.

Unless real-time data collection and processing is available (or specifically organised for the study), the time lag of RHCD should be acknowledged. For surveillance, methods other than RCHD are required for rapid safety reporting (e.g. 24‑h telephone service), especially for trials with longer follow-up.

## The Dutch Heart Failure registry: a case study

Currently, the NHR is setting up a heart failure registry [3, 25], which is partially based on the previous experience of the CHECK-HF (*Chronisch Hartfalen ESC-richtlijn Cardiologische praktijk Kwaliteitsproject HartFalen*) registry, which was conducted between 2013 and -2016 in 34 outpatient clinics in the Netherlands [26]. With the NHR Heart Failure registry, heart failure outpatient clinics can, on a voluntary basis, anonymously and periodically share a set of clinical parameters that reflect their clinical care. This set includes patient characteristics, the heart failure therapy received (drug classes and current dose, device therapy) and outcome measures (mortality, heart failure hospitalisations, quality of life). For the purposes of standardisation, the NHR provides a handbook with definitions; moreover, certified data quality control systems are in place to ensure the completeness and quality of data [27]. The NHR then provides feedback on the quality of care at an institutional level (comparison among peers). This may trigger further improvements, with the NHR as an integral part of a plan-do-check-act (PCDA) cycle. TITRATE-HF and associated trials (RELEASE-HF, ENGAGE-HF) are acting as accelerators in making it easier for institutes to join the NHR Heart Failure registry [28]. The 2021 NHR report mentions 13 contributing institutes, and since its initiation 1350 heart failure patients have been included in the registry [25]. For 2023, it is anticipated that > 30 institutes will participate.

**Fig. 2:**
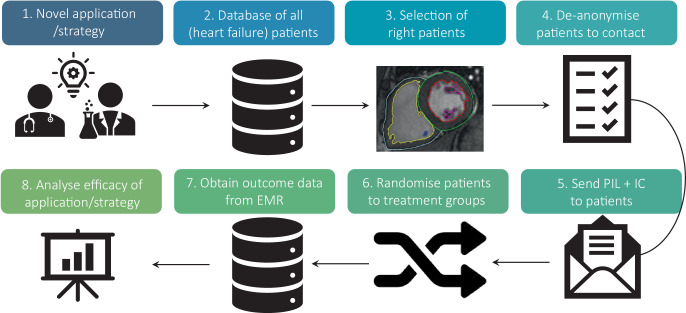
Key steps in registry-based randomised clinical trials. *PIL* patient information leaflet, *IC* informed consent, *EMR* electronic medical records

The first goal of the Dutch Heart Failure registry is to provide institutes with individual feedback on their quality of care in relation to other institutes, and to initiate a PDCA cycle. Secondly, national temporal trends can be described, e.g. the implementation of new heart failure guidelines. Ultimately, the registry must evolve into a nationwide data platform that is suitable for large, pragmatic, registry-based RCTs, with SELEQT-HF (selenium/CoQ10 supplementation in heart failure) as its first endeavour (Figs. 1 and 2).

**Fig. 3:**
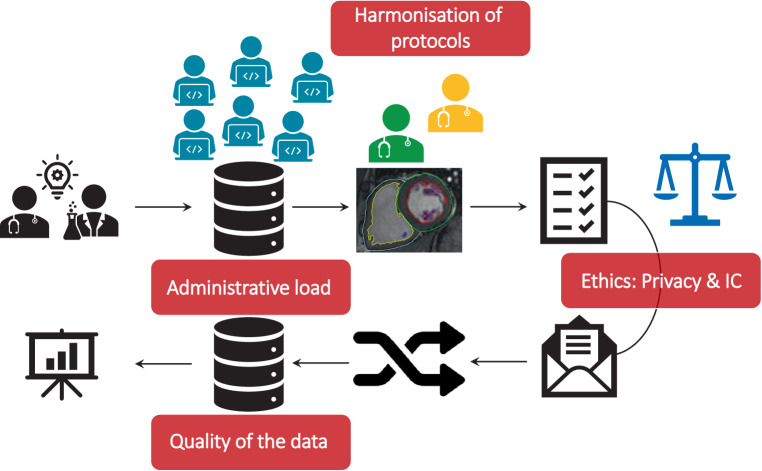
Major barriers that need to be overcome before registry-based, randomised clinical trials can be successfully conducted. *IC* informed consent

To make this initiative a success, certain barriers need to be overcome (Fig. 3; [29]). In order to be able to access the full potential of the data, it is important to embrace the opt-out approach for informed consent for observational studies. In order to achieve nationwide coverage, it is necessary to install multiple incentives for institutes to participate, possibly with central enforcement mechanisms, for example by making it a mandatory requirement by the professional associations (NVVC, NVT), as is already the case for percutaneous coronary interventions and cardiac surgery, or by payment per performance. In addition, registration should be made effortless and of high quality by the use of smart data extraction and standardisation of data acquisition in routine clinical practice, with a coordinating role for the NHR. Under these conditions, the ultimate goal of performing large pragmatic RCTs at low cost will come within reach.

## Conclusion

Large, well-executed RCTs remain the gold standard for clinical evidence, but they are complex, costly and slow. The use of RHCD in clinical trials may overcome these limitations. New European laws on privacy must prevent misuse of RCHD: obtaining informed consent by the opt-out method is a balanced way to perform large-scale observational studies that could contribute to the design of large pragmatic RCTs and improve recruitment. Linkage of data is challenging, requires extensive validation and the continuous efforts of many different stakeholders. Nevertheless, there are examples where RCHD sufficed for trial follow-up: the large size of the datasets compensated for underreporting and, by means of randomisation, observations were kept unbiased. For pharmacovigilance, the inherent delay when using RCHD should be acknowledged, and alternative methods for rapid safety reporting are essential. All things considered, the use of RHCD has immense potential, and while challenges exist, examples from the past have shown that these challenges can be overcome.
